# Ivermectin Strengthens Paclitaxel Effectiveness in High-Grade Serous Carcinoma in 3D Cell Cultures

**DOI:** 10.3390/ph18010014

**Published:** 2024-12-25

**Authors:** Mariana Nunes, Sara Ricardo

**Affiliations:** 1 Differentiation and Cancer Group, Institute for Research and Innovation in Health (i3S) of the University of Porto, 4200-135 Porto, Portugal; mnunes@i3s.up.pt; 2 Institute of Biomedical Sciences Abel Salazar (ICBAS), University of Porto, 4050-313 Porto, Portugal; 3 Associate Laboratory i4HB, Institute for Health and Bioeconomy, University Institute of Health Sciences (IUCS), University Polytechnic Higher Education Cooperative (CESPU), CRL, 4585-116 Gandra, Portugal; 4 Applied Molecular Biosciences Unit (UCIBIO), Toxicologic Pathology Research Laboratory, University Institute of Health Sciences (1H-TOXRUN, IUCS-CESPU), 4585-116 Gandra, Portugal; 5 Oral Pathology and Rehabilitation Research Unit (UNIPRO), Institute of Health Sciences (IUCS), Cooperativa de Ensino Superior Politécnico e Universitário (CESPU), Rua Central de Gandra 1317, 4585-116 Gandra, Portugal

**Keywords:** 3D cultures, chemoresistance, drug repurposing, high-grade serous carcinoma, drug synergism

## Abstract

Background: Chemoresistance is a major obstacle in high-grade serous carcinoma (HGSC) treatment. Although many patients initially respond to chemotherapy, the majority of them relapse due to Carboplatin and Paclitaxel resistance. Drug repurposing has surfaced as a potentially effective strategy that works synergically with standard chemotherapy to bypass chemoresistance. In a prior study, using 2D cultures and two HGSC chemoresistant cell lines, it was demonstrated that combining Carboplatin or Paclitaxel with Pitavastatin or Ivermectin resulted in the most notable synergy. Acknowledging that 2D culture systems are limited in reflecting the tumor architecture, 3D cultures were generated to provide insights on treatment efficacy tests in more complex models. Objectives: We aimed to investigate whether combining Carboplatin or Paclitaxel with Pitavastatin or Ivermectin offers therapeutic benefits in a Cultrex-based 3D model. Methods: Here, the cytotoxicity of Carboplatin and Paclitaxel, both alone and in combination with Pitavastatin or Ivermectin, were analyzed on two chemoresistant tumor cell lines, OVCAR8 and OVCAR8 PTX R C, in 3D cultures. Cellular viability was assessed using CellTiter-Glo^®^ Luminescent assays. Also, it explored synergistic interactions using zero interaction potency, Loewe, Bliss independence, and High-single agent reference models. Results: Our research indicates combining chemotherapeutic drugs with Pitavastatin or Ivermectin yields significantly more cytotoxic effects than chemotherapy alone. For all the combinations tested, at least one model indicated an additive effect; however, only the combination of Paclitaxel and Ivermectin consistently demonstrated an additive effect across all chemoresistant cell lines cultured in 3D models, as well as in all four synergy reference models used to assess drug interactions. Conclusions: Combining Paclitaxel with Ivermectin has the highest cytotoxic and the strongest additive effect for both chemoresistant cell lines compared to Paclitaxel alone.

## 1. Introduction

The standard treatment for HGSC includes Carboplatin and Paclitaxel; however, these can lead to chemoresistance and adverse side effects, affecting treatment effectiveness [1,2,3,4]. Combining repurposed drugs with chemotherapy may enhance efficacy, reduce toxicity, and counter-resistance [5,6,7]. This approach investigates new applications for existing approved drugs, potentially saving time and costs in drug development [5,6,7]. Since these drugs have established pharmacological and toxicological profiles, the approval for new indications is typically faster than for new drugs [8,9,10]. However, appropriate clinical trials must be established to integrate repurposed drugs into oncology, assess drug efficacy, and determine the maximum tolerated dose to avoid intolerable toxicities [8].

Addressing chemoresistance can involve combining drugs at optimal ratios to target different mechanisms, enhancing sensitivity and efficacy [11]. Interactions can yield synergistic, additive, or antagonistic effects, with synergy offering the greatest benefit by surpassing individual activities [12]. Antagonism reduces effectiveness, while additivity results in a combined impact equal to the sum. Synergistic use often allows for lower dosages, decreasing systemic toxicity and side effects [13,14,15].

Pitavastatin, an antilipidemic drug, may benefit cancer therapy through its anti-tumoral effects, reduction of inflammation, and synergistic effects with other drugs [6]. Statins inhibit the mevalonate pathway, impacting cell proliferation and survival, potentially slowing tumor growth, and enhancing other treatments [16]. Some studies suggest combining statins with chemotherapy improves outcomes by increasing cancer cell sensitivity [17,18]. Statins’ anti-inflammatory properties may also reduce the supportive tumor microenvironemnt (TME) [19].

Ivermectin, an antiparasitic agent, may have anti-cancer properties [6]. In some cases, it has been found to enhance the effectiveness of chemotherapy drugs and even reverse resistance [17,20]. Ivermectin can act as a chemosensitizer by blocking drug efflux, increasing drug accumulation, and improving the efficacy of antineoplastic drugs [6,21].

Our previous studies using 2D models indicated that Pitavastatin and Ivermectin significantly decreased the cellular viability of OVCAR8 and OVCAR8 PTX R P cells [17]. In contrast, both drugs showed minimal effect on the viability of HOSE6.3, a normal-like cell line, highlighting their differential impact on malignant versus normal-like cells [17]. This suggests that Pitavastatin and Ivermectin maintain a favorable safety profile in normal-like cells while exhibiting substantial anticancer efficacy in chemoresistant tumor cells, positioning them as promising candidates for combination therapy with Carboplatin and Paclitaxel [17,18]. Moreover, the results showed that combining Carboplatin or Paclitaxel with Pitavastatin in 2D models had the strongest synergistic cytotoxic effect on the chemoresistant cancer cells, surpassing the impact of chemotherapeutic drugs alone [17,18]. The same effect was also seen for the combination of Paclitaxel with Ivermectin in 2D models [17]. Although the synergistic effects of combinations such as Carboplatin or Paclitaxel with Pitavastatin and Paclitaxel with Ivermectin have been demonstrated in 2D cultures, the transition to Three-dimensional (3D) models is crucial. 3D cell cultures more accurately mimic the complexity of the TME, including factors like cell-cell interactions, nutrient gradients, and drug diffusion, which are not represented in 2D cultures. By testing these drug combinations in a 3D model, we are better able to assess their real-world potential for overcoming chemoresistance, as these models provide a more clinically relevant environment for drug testing.

It is essential to recognize that 2D cell culture models often fail to accurately mimic the complex architecture *in vivo* environments found in living organisms. These models can oversimplify interactions between cells and their microenvironments, leading to results that may not effectively translate to clinical scenarios [22,23,24]. Researchers have developed 3D cell culture technologies to address these limitations, which provide a more realistic representation of tissue architecture and cellular interactions. Moreover, drugs have more difficulty reaching the cells when they are in a 3D structure, as the increased complexity and density of the 3D microenvironment can impede drug penetration and lead to uneven distribution, in contrast to the more accessible and uniform environment of 2D cell cultures [25,26,27].

Therefore, it possible to conduct drug studies with greater precision using 3D models, allowing for more reliable insights into how treatments may perform in actual biological systems [22,23,24].

Here, we investigated whether combining Carboplatin or Paclitaxel with Pitavastatin or Ivermectin provided therapeutic benefits in a Cultrex^TM^-based 3D cell model using two chemoresistant HGSC cell lines (OVCAR8 and OVCAR8 PTX R C).

Although at least one of the reference models indicated an additive effect for all the combinations tested, only the combination of Paclitaxel and Ivermectin demonstrated a consistent additive effect across all chemoresistant cell lines cultured in 3D models and in all four synergy reference models used to assess drug interactions. In summary, our findings demonstrate that combining Paclitaxel with Ivermectin has the highest cytotoxic and strongest additive effect for both chemoresistant cell lines compared to Paclitaxel alone.

## 2. Results

### 2.1. Combining Carboplatin with Pitavastatin or Ivermectin Has an Additive Effect on OVCAR8 and OVCAR8 PTX R C Cells

This study utilized a predefined combination model to combine Carboplatin with Pitavastatin or Ivermectin. Chemoresistant HGSC cells were plated in 3D culture and treated with each drug individually and in combination at set ratios based on their IC_50_ values. The findings indicated that for OVCAR8, the combination of Carboplatin and Pitavastatin significantly enhanced the anticancer effect (*p* < 0.0001) at 0.25, 0.5, 1, and 2 times the individual IC_50_ values, compared to Carboplatin alone (Figure S1A). For OVCAR8 PTX R C cells, this combination significantly increased the anticancer effect (*p* < 0.0001) for 0.25, 0.5, and 1 times the individual IC_50_ values compared to Carboplatin as a single agent (Figure S1B).

For OVCAR8, combining Carboplatin with Ivermectin significantly enhanced the anticancer effect (*p* < 0.0001) at 0.25, 0.5, and 1 times the IC_50_ values, in contrast to Carboplatin alone (Figure S1C). For OVCAR8 PTX R C cells, this combination also heightened the antineoplastic effect at 0.25 (*p* < 0.001) and 0.5 (*p* < 0.0001) times the IC_50_ values, compared to Carboplatin as a single agent (Figure S1D).

The synergistic effect is the observed effect exceeding the expected effect derived from reference models (synergy scoring models). Each model operates under different assumptions about the predicted effect due to their unique mathematical frameworks [28]. With this in mind, four distinct methods—zero interaction potency (ZIP), Loewe, Bliss independence, and high-sigle agent (HSA)—were employed to evaluate drug interactions and compare the outcomes. The ZIP model calculates the expected effect based on the premise that the two drugs do not enhance each other [29]. The Loewe additivity model defines the expected effect *yLOEWE* as if a drug were combined with itself and incorporates the dose-response curves of each drug [30]. The Bliss model operates on the assumption of a stochastic process where two drugs independently exert their effects, allowing for the calculation of the expected combination effect based on the probabilities of independent events [31]. HSA asserts that the expected combination effect is equal to the greater effect of the individual drugs [32]. In all four models, the synergy score for a drug combination is averaged over all the dose combination measurements, resulting synergy score values: >10 (synergy, red) and <−10 (antagonism, green) [33,34].

**Figure 1:**
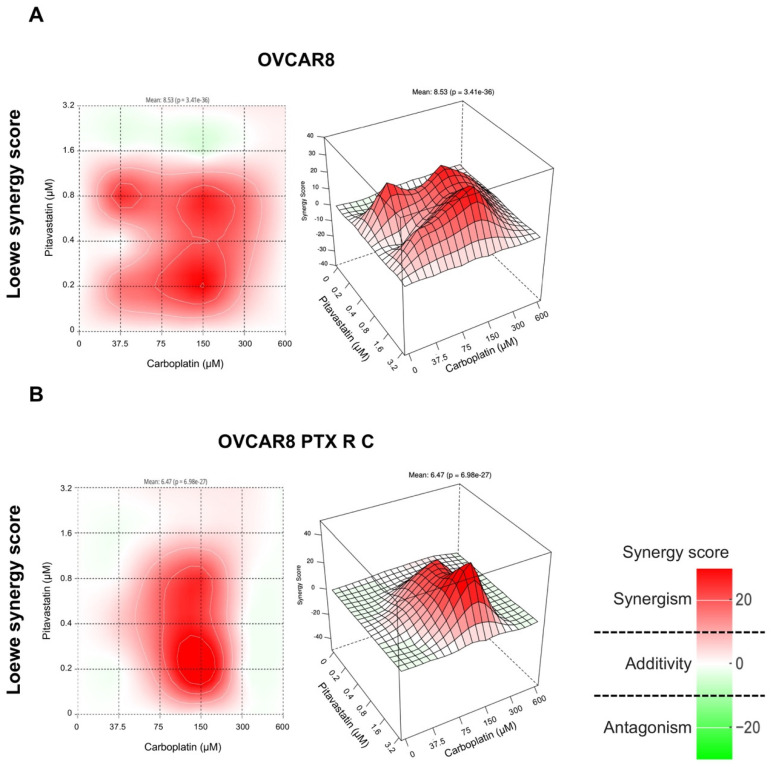
Combining Carboplatin with Pitavastatin has an additive effect on OVCAR8 and OVCAR8 PTX R C cells according to the Loewe synergy model. Two- and three-dimensional synergy plots showed the drug synergism of OVCAR8 (**A**) and OVCAR8 PTX R C (**B**) cells after exposure to fixed-dose ratios that correspond to 0.25, 0.5, 1, 2, and 4 times the individual IC_50_ values of each drug. Carboplatin was combined with Pitavastatin for 72 h. The combined treatment was administered simultaneously. All assays were performed in triplicate in at least three independent experiments. The synergy scores were categorized as follows: <−10 (antagonism, green), −10 to 10 (additivity, white), and >10 (synergism, red). HSA, high single agent; ZIP, zero interaction potency.

Findings from the analysis of OVCAR8 cells for three of the four models used to assess drug interactions revealed negative synergy scores of −41.19, −45.56, and −43.36 when combining Carboplatin with Pitavastatin, indicating antagonism (green) (Figure S2A–C). Yet, the Loewe model shows a positive synergy score of 8.53, suggesting additivity (white) along with some synergistic zones (red) for these combinations at lower and intermediate concentrations (Figure 1A). In the case of OVCAR8 PTX R C cells, three of the four models used to assess drug interactions yielded negative synergy scores of −45.77, −48.63, and −47.62, indicating antagonism (green) (Figure S2D–F). Nonetheless, the Loewe model reported a positive synergy score of 6.47, suggesting additivity (white) and some synergistic zones (red) for the combinations at lower to intermediate concentrations (Figure 1B).

**Figure 2:**
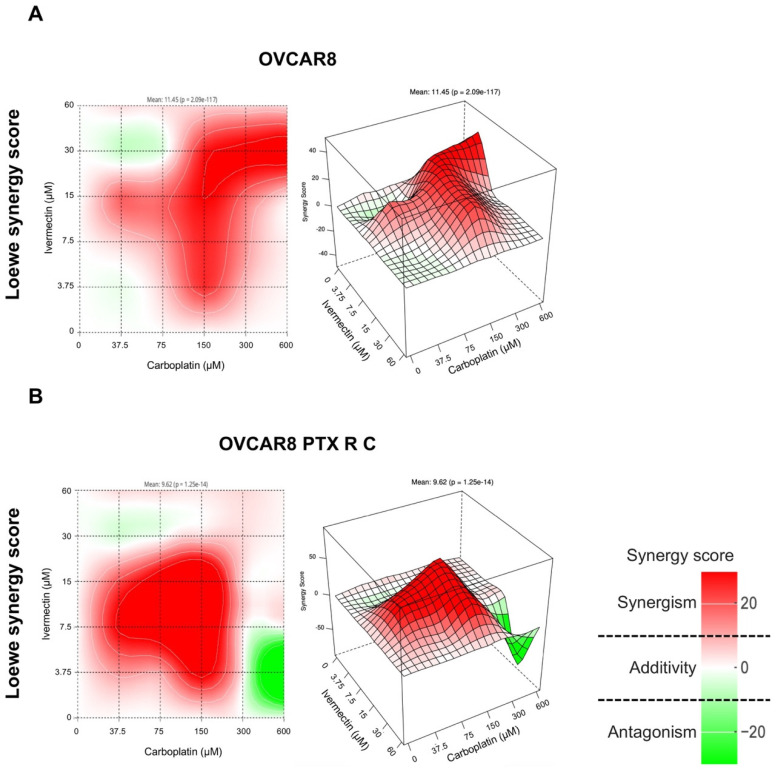
Combining Carboplatin with Ivermectin has an additive effect on OVCAR8 and OVCAR8 PTX R C cells according to the Loewe synergy model. Two- and three-dimensional synergy plots showed the drug synergism of OVCAR8 (**A**) and OVCAR8 PTX R C (**B**) cells after exposure to fixed-dose ratios that correspond to 0.25, 0.5, 1, 2, and 4 times the individual IC_50_ values of each drug. Carboplatin was combined with Ivermectin for 72 h. The combined treatment was administered simultaneously. All assays were performed in triplicate in at least three independent experiments. The synergy scores were categorized as follows: <−10 (antagonism, green), −10 to 10 (additivity, white), and >10 (synergism, red). HSA, high single agent; ZIP, zero interaction potency.

For OVCAR8 cells, the findings for three of the four models used to assess drug interactions indicate negative synergy scores of −37.65, −42.89, and −42.47 when combining Carboplatin with Ivermectin, confirming antagonism (green) (Figure S3A–C). Additionally, the Loewe model indicated a positive synergy score of 11.45, suggesting synergy (red) (Figure 2A). Regarding OVCAR8 PTX R C cells, this combination for three of the four models used to assess drug interactions showed negative synergy scores of −32.70, −38.80, and −27.68, indicating antagonism (green) (Figure S3D–F). Furthermore, the Loewe model reported a positive synergy score of 9.62, suggesting additivity (white) along with some synergistic zones (red) for the combinations at lower and intermediate concentrations (Figure 2B).

### 2.2. Combining Paclitaxel with Pitavastatin or Ivermectin Has an Additive Effect on OVCAR8 and OVCAR8 PTX R C Cells

We also studied the combination of Paclitaxel with Pitavastatin or Ivermectin using the same 3D culture and drug combination model. The findings indicate that in OVCAR8 cells, the combination of Carboplatin and Pitavastatin significantly boosted the anticancer effect at 0.25 (*p* < 0.05) and 0.5, 1, and 2 (*p* < 0.0001) times the individual IC_50_ values in contrast to Paclitaxel alone (Figure S4A). In OVCAR8 PTX R C cells, this combination markedly enhanced anticancer efficacy (*p* < 0.0001) at all five IC_50_ values compared to Paclitaxel as a single agent (Figure S4B).

For OVCAR8, the combination of Paclitaxel with Ivermectin also resulted in a significant increase in anticancer effects at the tested concentrations (*p* < 0.01 for 0.25 and *p* < 0.0001 for 0.5, 1, and 2 times the IC_50_ values) compared to Paclitaxel alone (Figure S4C). In OVCAR8 PTX R C cells, this combination yielded a notable enhancement in anti-tumor effects (*p* < 0.0001) for all five IC_50_ values compared to Paclitaxel alone (Figure S4D).

**Figure 3:**
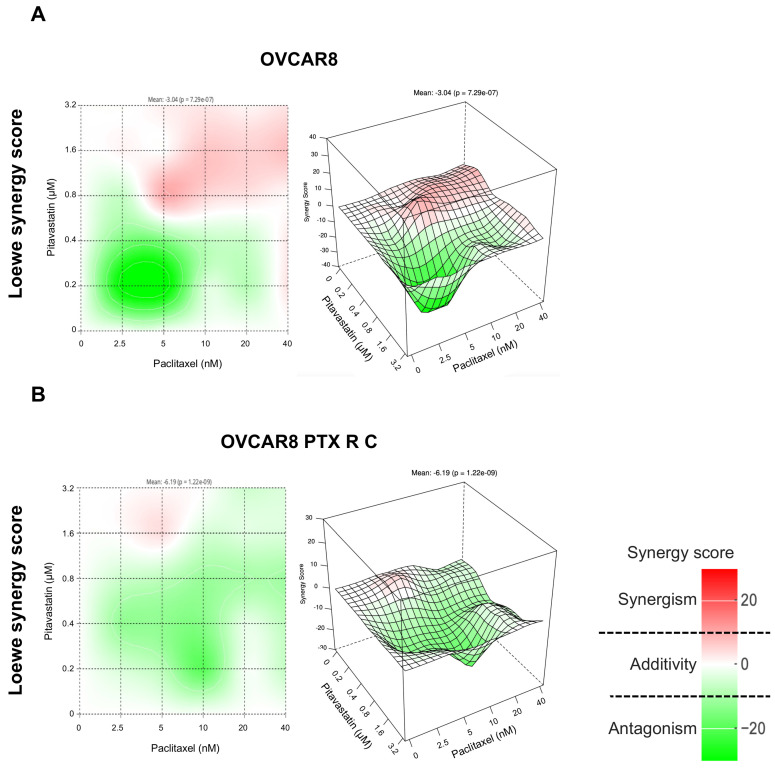
Combining Paclitaxel with Pitavastatin has an additive effect on OVCAR8 and OVCAR8 PTX R C cells according to the Loewe synergy model. Two- and three-dimensional synergy plots showed the drug synergism of OVCAR8 (**A**) and OVCAR8 PTX R C (**B**) cells after exposure to fixed-dose ratios that correspond to 0.25, 0.5, 1, 2, and 4 times the individual IC_50_ values of each drug. Paclitaxel was combined with Pitavastatin for 72 h. The combined treatment was administered simultaneously. All assays were performed in triplicate in at least three independent experiments. The synergy scores were categorized as follows: <−10 (antagonism, green), −10 to 10 (additivity, white), and >10 (synergism, red). HSA, high single agent; ZIP, zero interaction potency.

In this study involving OVCAR8 cells across four models used to assess drug interactions, it was found that combining Paclitaxel with Pitavastatin yielded synergy scores of 1.00, −3.04, −6.44, and −5.16, suggesting an additive effect (white). Notably, both reference models also identified synergic zones (red) at intermediate to higher concentrations (Figure 3A and Figure S5A–C). For OVCAR8 PTX R C, the combination of Paclitaxel and Pitavastatin showed HSA and Loewe model synergy scores of −3.13 and −6.19, respectively, indicating additivity (white). In contrast, Bliss independence and ZIP models revealed scores of −11.96 and −11.28, respectively, indicating antagonism (green) (Figure 3B and Figure S5D–F).

**Figure 4:**
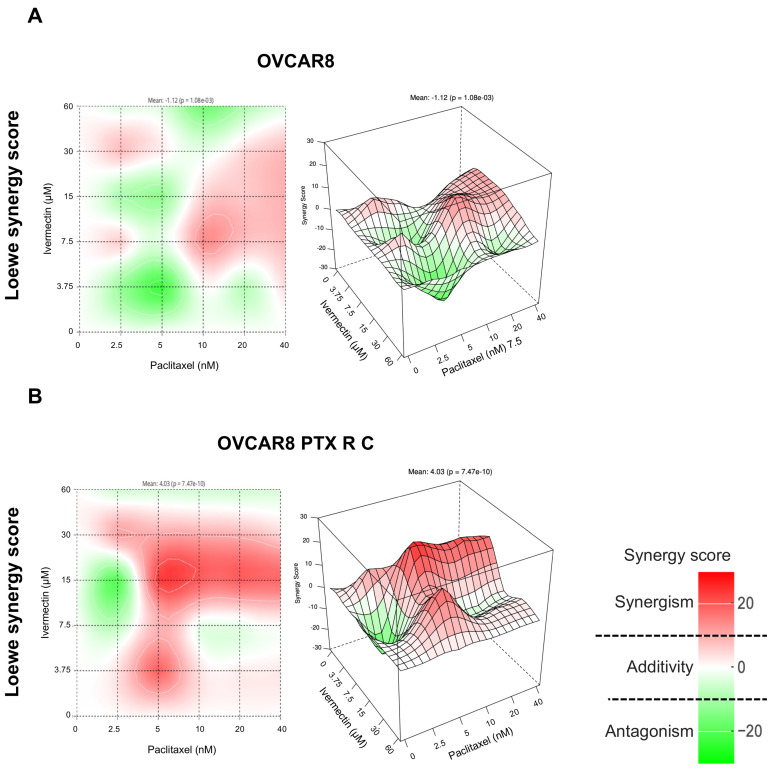
Combining Paclitaxel with Ivermectin has an additive effect on OVCAR8 and OVCAR8 PTX R C cells according to the Loewe synergy model. Two- and three-dimensional synergy plots showed the drug synergism of OVCAR8 (**A**) and OVCAR8 PTX R C (**B**) cells after exposure to fixed-dose ratios that correspond to 0.25, 0.5, 1, 2, and 4 times the individual IC_50_ values of each drug. Paclitaxel was combined with Ivermectin for 72 h. The combined treatment was administered simultaneously. All assays were performed in triplicate in at least three independent experiments. The synergy scores were categorized as follows: <−10 (antagonism, green), −10 to 10 (additivity, white), and >10 (synergism, red). HSA, high single agent; ZIP, zero interaction potency.

Conversely, the combination of Paclitaxel and Ivermectin indicated additivity (white), with synergy scores of 5.15, −1.12, −1.28, and −0.86 for OVCAR8 cells (Figure 4A and Figure S6A–C). All four reference models used to assess drug interactions also indicated synergic zones (red) at low, intermediate, and higher concentrations. For OVCAR8 PTX R C cells, this combination reflected an additive (white) effect as well, with synergy scores of 8.21, 4.03, 0.12, and 0.23 (Figure 4B and Figure S6D–F), but also showing synergic zones (red) at low to intermediate concentrations. These findings underscore that different synergy evaluation models can yield varying scores; however, all four methods demonstrated consistency in results for the combinations tested. Thus, it is evident that the combination of Paclitaxel and Ivermectin is the most promising combination for both HGSC cell lines.

### 2.3. Morphological Assessment Reveals Drug Efficacy in Combined Treatments

**Figure 5:**
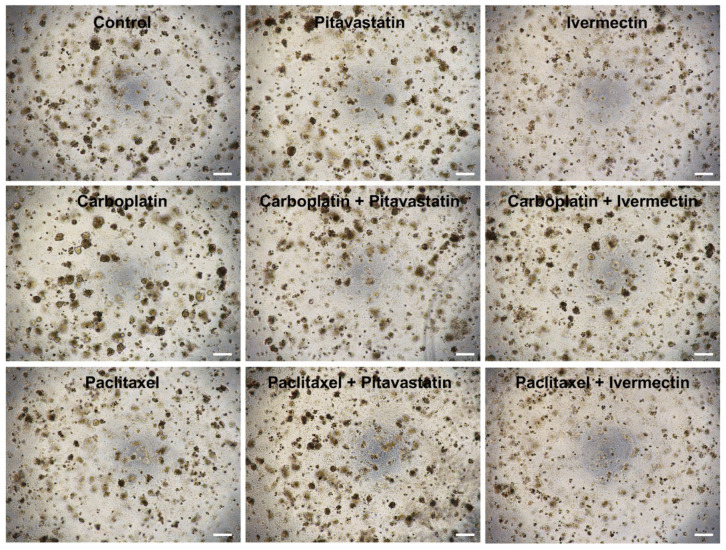
Pitavastatin and Ivermectin improved the cytotoxic effect of Carboplatin and Paclitaxel on OVCAR8 cells. Representative microscopy images of OVCAR8 cells after exposure for 72 h to control Carboplatin, Paclitaxel, Pitavastatin, Ivermectin, Carboplatin + Pitavastatin, Carboplatin + Ivermectin, Paclitaxel + Pitavastatin and Paclitaxel + Ivermectin at IC_50_ values of each drug (Carboplatin 150 μM, Paclitaxel 10 nM, Pitavastatin 0.8 μM and Ivermectin 15 μM). All assays were performed in triplicate in at least three independent experiments. Scale bar, 200 μm. HGSC, high-grade serous carcinoma.

**Figure 6:**
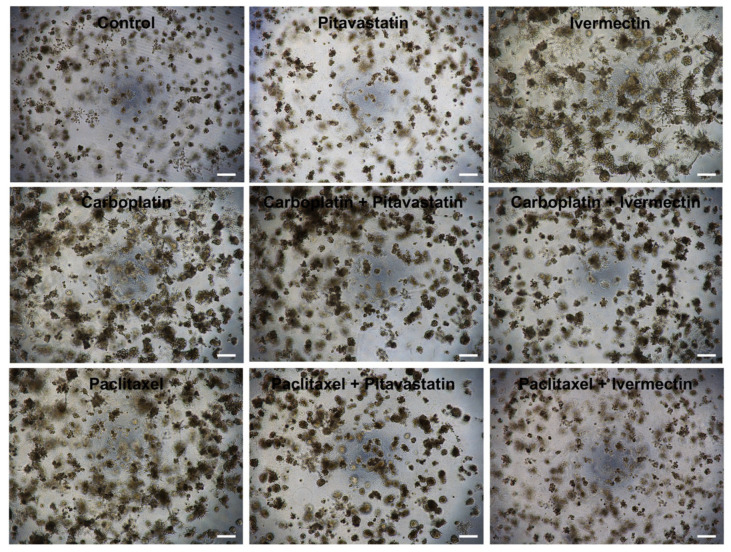
Pitavastatin and Ivermectin improved the cytotoxic effect of Carboplatin and Paclitaxel on OVCAR8 PTX R C cells. Representative microscopy images of OVCAR8 PTX R C cells after exposure for 72 h to control, Carboplatin, Paclitaxel, Pitavastatin, Ivermectin, Carboplatin + Pitavastatin, Carboplatin + Ivermectin, Paclitaxel + Pitavastatin and Paclitaxel + Ivermectin at IC_50_ values of each drug (Carboplatin 150 μM, Paclitaxel 10 nM, Pitavastatin 0.8 μM and Ivermectin 15 μM). All assays were performed in triplicate in at least three independent experiments. Scale bar, 200 μm. HGSC, high-grade serous carcinoma.

We also performed a morphological assessment for each treatment scenario (see Figure 5 and Figure 6) since live and dead cells in 3D cultures exhibit distinct characteristics, including cellular morphology, proliferation, metabolic activity, cell-cell interactions, and membrane integrity. Recognizing these differences is essential for understanding how cells respond to treatments in 3D cultures, offering valuable insights into drug efficacy.

Our results align with previous findings, showing morphological differences in OVCAR8 and OVCAR8 PTX R C cells when subjected to single and combined treatments, as opposed to the control. When Carboplatin or Paclitaxel was combined with Pitavastatin or Ivermectin at IC_50_ values, it induced a more aggressive phenotype, leading to a range of observable characteristics in dead cells within 3D conformation, including decreased cell numbers, reduced aggregate formation, and the presence of smaller, rounded cells (Figure 5 and Figure 6). Additionally, signs of disrupted cellular structures, such as fragmented nuclei and loss of membrane integrity, were observed.

The control for OVCAR8 and OVCAR8 PTX R C cell lines displayed live cells that typically retain a healthy, elongated, or polygonal shape, mirroring their natural tissue architecture. Active mitosis occurs, evidenced by distinct nuclei and clear boundaries between cells. High metabolic activity is indicated by nutrient uptake and the production of metabolic byproducts, as seen in the color saturation of the medium. Robust intercellular connections and signaling foster tissue-like organization and functionality, with cells closely aggregated and connected and intact cell membranes that prevent cytoplasmic leakage (Figure 5 and Figure 6).

In contrast, OVCAR8 and OVCAR8 PTX R C cells subjected to combined treatments show visibly dead cells that are typically smaller and rounded, accompanied by a loss of structural integrity. The notable reduction in cell density resulting from cell death affects aggregate formation as a whole. This includes indications of nuclear fragmentation and chromatin condensation, suggesting apoptosis or necrosis. Additionally, low or absent metabolic activity is reflected in diminished nutrient uptake and a lack of ATP production (Figure 5 and Figure 6).

## 3. Discussion

Evidence suggests that combining chemotherapeutic agents with repurposed drugs can enhance therapeutic efficacy by targeting different mechanisms or pathways in a synergistic or additive way [35,36,37,38]. Preclinical and retrospective studies have identified Pitavastatin and Ivermectin as potential antineoplastic compounds [20,39,40,41,42,43,44]. In earlier research using 2D cell cultures, both drugs emerged as promising candidates for enhancing the effectiveness of Carboplatin or Paclitaxel in OVCAR8 and OVCAR8 PTX R P cells [17,18].

In the current study, we investigated the combined effect of Carboplatin or Paclitaxel with Pitavastatin or Ivermectin on Carboplatin-resistant (OVCAR8) and Carboplatin–Paclitaxel-resistant (OVCAR8 PTX R C) cell lines under 3D culture conditions. Both cell lines were exposed to varying concentrations (0.25-, 0.5-, 1-, 2-, and 4-fold the IC_50_ values) of each drug alone and in combination with the chemotherapeutic agents. The results indicated that Ivermectin is the most effective repurposed drug in improving the performance of Paclitaxel in both chemoresistant cell lines under 3D culture conditions. This supports the potential of combining Paclitaxel with repurposed drugs, warranting further investigation to evaluate its impact on patient-derived cells with chemoresistant disease.Notably, 3D cultures are increasingly recognized for their relevance in drug testing and cancer research. These models provide a more accurate representation of tumor characteristics, such as cellular interactions with stromal cells and extracellular matrix (ECM) components, which are important for understanding metastasis and drug efficacy [23,45,46]. Additionally, 3D cultures facilitate high-throughput screening that yields more relevant data on drug efficacy and toxicity compared to traditional 2D models [45]. They also support personalized medicine, allowing the evaluation of drug responses in patient-derived cultures to tailor treatment strategies [47].

The efficacy of drugs in 2D cultures may differ significantly from their effects in 3D environments. In 2D models, cells are arranged in a single layer, allowing for more uniform drug diffusion, which can lead to higher effective drug concentrations and more pronounced responses [25,26,27]. Conversely, in 3D cultures, drug penetration can be hindered, especially for cells located deeper within the structure, potentially resulting in a lower effective drug concentration and a slower or less pronounced response [25,26,27]. However, further experimental data are needed to determine whether the reduced efficacy observed in 3D cultures is due to lower drug concentrations or different cellular responses in these two models.

Although 3D *in vitro* models can represent some features of tumor architecture, they have considerable limitations, especially in recreating the systemic factors that affect drug distribution, metabolism, and effectiveness [46,48]. Important components like tumor vascularization, the immune response, and the interaction between the tumor and surrounding tissues are challenging to fully replicate in cell culture models [46,48]. These factors are vital for comprehending how treatments are distributed and metabolized in the body, directly influencing therapeutic outcomes. This issue is particularly crucial when trying to translate preclinical results into clinical contexts where the patient’s condition is subject to a more intricate physiological landscape.

We recognize this limitation and agree that a significant challenge in cancer research is achieving optimal drug concentrations at the tumor site *in vivo*. This concentration is vital for therapeutic effectiveness, especially for drugs that must navigate complex biological barriers, such as the ECM and the altered networks of blood vessels found in tumors.

To address these limitations, future research should employ more sophisticated models that accurately reflect human physiology. Systems like patient-derived organoids and patient-derived xenografts show promise in this area, as they facilitate a more accurate depiction of tumor interactions with the systemic environment, encompassing immune responses, blood flow dynamics, and vascular structure [49,50]. Implementing these models could enhance our understanding of the TME factors affecting drug action and allow for a more precise evaluation of drug efficacy and safety, bridging the gap between preclinical findings and clinical results. Controlling drug concentrations *in vivo* is crucial for potential synergistic effects. Optimal dosing and timing significantly impact efficacy, making control essential for translating preclinical findings to clinical outcomes. Achieving synergy requires precise concentration control and understanding of the pharmacokinetics and pharmacodynamics of each agent. This involves optimizing drug administration timing for effective collaboration. Advanced modeling techniques, like pharmacokinetic/pharmacodynamic modeling, can predict optimal dosing schedules and concentrations for combinations, aiding in effective *in vivo* experiments. Ultimately, achieving the right drug concentration balance while minimizing adverse effects needs a multidisciplinary approach, incorporating advanced drug delivery technologies and precise dosing strategies alongside a deep understanding of tumor biology and TME [51,52]. Future studies must incorporate these considerations to maximize therapeutic potential in clinical settings.

A key challenge in overcoming chemoresistance is the reduced accumulation of chemotherapeutic agents in resistant cells. This is often due to altered transporter expression, which reduces intracellular drug levels, thus mitigating cytotoxic effects [53]. In particular, P-gp, an efflux transporter, plays a critical role in MDR by actively exporting various chemotherapeutic agents, such as Paclitaxel, from cells, thereby decreasing their intracellular concentrations [54]. It has been shown that OVCAR8 PTX R C cells, which are resistant to Paclitaxel, exhibit elevated P-gp expression [55]. In this study, OVCAR8 PTX R C cells demonstrated that Ivermectin, when combined with Paclitaxel, significantly increased drug efficacy, particularly in synergistic areas of the response curve.

Ivermectin has been shown to interact with P-gp both as a substrate and a modulator, inhibiting its efflux function and thereby increasing intracellular drug levels [56,57]. This interaction is significant in overcoming chemoresistance, as P-gp is involved in tumor cells’ resistance to chemotherapy. By modulating P-gp activity, Ivermectin has the potential to enhance the effectiveness of existing chemotherapeutic treatments, making it a promising candidate for combination therapies aimed at addressing MDR in cancer. However, the impact of Ivermectin on P-gp modulation may vary depending on the model system used. In 2D cultures, the lack of cellular interactions and ECM components may facilitate a more direct impact on P-gp activity, potentially resulting in higher drug concentrations within cells. In contrast, 3D cultures present more complex environments, where cellular interactions, nutrient gradients, and the ECM may limit drug penetration and distribution. Consequently, higher drug concentrations may be needed in 3D models to achieve similar effects on P-gp expression and activity as seen in 2D cultures.

When we compare the results from this study with our earlier work, which used 2D cultures of HGSC chemoresistant cells [17,18], fewer synergistic pairs were observed in Cultrex^TM^-based 3D models. This difference may be related to variations in P-gp expression between the two model systems. Studies have shown that P-gp behavior differs significantly between 2D and 3D cultures, influencing both drug effectiveness and resistance [58]. Future studies will monitor P-gp activity in 3D cultures using flow cytometry and fluorescent substrates (e.g., RH-123) or confocal microscopy to assess P-gp expression, efflux activity, and localization within these models. These experiments will provide crucial insights into the role of P-gp in resistance and help to develop more effective cancer therapies.

In 3D settings, cells interact in a more physiologically relevant manner, which can alter P-gp expression and function [59,60,61]. These interactions, along with the physical and chemical gradients present in 3D models, can influence drug resistance mechanisms. For example, growth factors, cytokines, and intercellular communication in 3D cultures can promote or reduce P-gp expression and activity [59,60,61]. The cytoskeletal structure, which is critical for the localization and function of P-gp, may also be more complex in 3D models, potentially affecting how P-gp transports drugs to the cell membrane [62]. Additionally, the structural characteristics of 3D cultures, such as spheroid formation and scaffold materials, can affect drug penetration and distribution, leading to differential drug exposure and altering P-gp-mediated efflux.

To gain further insight into P-gp behavior and its impact on drug resistance, future research will focus on monitoring P-gp activity in 3D cultures, evaluating the role of P-gp inhibitors, and exploring how these interactions may improve the effectiveness of Paclitaxel and other chemotherapy drugs in resistant HGSC. Investigating P-gp in 3D models will offer valuable information on overcoming resistance in OC and lead to more effective combination therapies. Still, these findings should be interpreted with caution, and that further validation in more complex models is needed.

In conclusion, this study’s findings suggest that compounds like Ivermectin may hold significant potential as chemosensitizers in the treatment of chemoresistant HGSC. By modulating P-gp and other transport proteins, Ivermectin could enhance the cytotoxicity of platinum-based therapies, overcoming resistance and improving patient outcomes. Further research is needed to clarify the mechanisms underlying these interactions and explore the clinical applicability of Ivermectin and other repurposed drugs in combination with chemotherapy.

## 4. Materials and Methods

### 4.1. Cell Lines and Culture Conditions

The OVCAR8 (resistant to Carboplatin) [35] and OVCAR8 PTX R C (resistant to both Carboplatin and Paclitaxel) [55] cell lines were chosen as models for HGSC. Further information regarding the cell lines and their culture conditions has been provided previously [17].

### 4.2. Drugs

Carboplatin, Paclitaxel, Pitavastatin, and Ivermectin were acquired from Selleckchem (Houston, TX, USA). They were dissolved in DMSO (AppliChem, Barcelona, Spain) or distilled water and stored at −80 °C according to the manufacturer’s guidelines. Before usage, an aliquot was diluted to the necessary concentrations.

### 4.3. Cell Viability Assay

We employed CellTiter-Glo^®^ luminescent assays to assess cellular viability following the manufacturer’s instructions (Promega Corporation, Madison, WI, USA). For creating and sustaining stable 3D dome structures on cell culture plates, Cultrex^TM^ Reduced Growth Factor Basement Membrane Extract, Type 2 (Bio-Techne, Minneapolis, MN, USA) was utilized. First, it was started by preparing a cell suspension with 2.5 × 10^3^ cells per well in complete media with 60% Cultrex^TM^. Next, 10 µL of this suspension was carefully added into each well of a 96-well black/clear bottom plate and incubated at 37 °C with 5% CO_2_ for 2 min. Afterward, the plate was inverted and incubated at 37 °C with 5% CO_2_ for 25 min to ensure full Cultrex^TM^ polymerization. Then, a complete medium was added and incubated under the same conditions. After 96 h, the cells were treated with escalating concentrations of Carboplatin (37.5 to 600 µM), Paclitaxel (2.5 to 40 nM), Pitavastatin (0.2 to 3.2 µM), and Ivermectin (3.75 to 60 µM), in a final volume of 100 µL per well. The range of concentrations used in this analysis was previously determined and applied in the 2D models [17,18]. The IC_50_ values for these drugs were derived from experiments with the OVCAR8 cell line and were as follows: 150 µM for Carboplatin, 10 nM for Paclitaxel, 0.8 µM for Pitavastatin, and 15 µM for Ivermectin [17]. The cells were then incubated under the same conditions for an additional 72 h. Following this was discarded the medium and introduced 50 µL of CellTiter-Glo^®^ 2.0 Reagent (Promega Corporation, Madison, WI, USA), diluted 1:1 in culture medium, allowing it to incubate for 10 min at RT, shielded from light to ensure the stability of the luminescent signal. Luminescence was then measured using a Bio Tek Synergy™ 2 multi-mode microplate reader (BioTek, Winooski, VT, USA). The treated cells were compared to control cells, which were considered 100% viable, containing 1% (*v*/*v*) of the vehicle (DMSO), with white wells (without cells) as the baseline.

### 4.4. Drug Treatment and Interaction Analysis

Earlier reports presented the IC_50_ values for each drug [17,55]. These values were utilized in combination studies, which involved varying concentrations of both drugs in a consistent ratio, as outlined in previous methodologies [34]. Specifically, Carboplatin or Paclitaxel was combined with various repurposed drugs at fixed-dose ratios corresponding to 0.25, 0.5, 1, 2, and 4 times the individual IC_50_ values over 72 h.

This research further examined the responses to drug combinations using SynergyFinder Plus Software 3.0 (Netphar, Faculty of Medicine, University of Helsinki, Helsinki, Finland). This software facilitated the estimating of expected drug combination effects based on the zero interaction potency, Loewe, Bliss independence, and HSA reference models [63,64]. It enables interactive analysis and visualization of multidrug combination profiling, showcasing synergy (in red) and antagonism (in green) on both 2D and 3D synergy maps [33,34]. Additionally, the cNMF algorithm within SynergyFinder Plus Software was employed to identify outlier measurements [65].

### 4.5. Microscopic Evaluation

All microscopic figures were captured under a Leica DMi1 inverted phase contrast microscope (Leica Microsystems, Wetzlar, Germany) at 50× magnification.

### 4.6. Statistical Analysis

All assays were performed in triplicate with at least three independent experiments. The data were expressed as the mean ± SD. Statistical analysis was performed in GraphPad Prism 10 (GraphPad Software Inc., Boston, CA, USA) using ordinary one-way or two-way ANOVA followed by Šıdák’s multiple comparison test.

## 5. Conclusions

3D cell culture models represent more reliable models in cellular biology and biomedical research. By offering a more precise representation of *in vivo* architecture, these models deepen understanding of intricate biological processes.

Our results showed that for all the combinations tested, at least one synergy reference model indicated an additive effect; however, only the combination of Paclitaxel and Ivermectin consistently demonstrated an additive effect across all chemoresistant cell lines cultured in a Cultrex^TM^-based 3D cell culture model, as well as in all four synergy reference models. Combining Paclitaxel with Ivermectin has the highest cytotoxic and the strongest additive effect for both chemoresistant cell lines compared to Paclitaxel alone. Paclitaxel’s clinical effectiveness can be reduced due to its active removal from cells through P-gp. Ivermectin acts as both a substrate and modulator of P-gp, attaching to this protein and making it less accessible. This relationship can increase the effectiveness of chemotherapy drugs. Thus, Ivermectin may function as a supportive addition to Paclitaxel treatment, enhancing therapeutic effectiveness, reducing toxicity associated with Paclitaxel, and helping to overcome chemoresistance.

## Data Availability

Data is contained within the article or Supplementary Material.

## Supplementary Materials

The following supporting information can be downloaded at: https://www.mdpi.com/article/10.3390/ph18010014/s1, Figure S1: Pitavastatin and Ivermectin increase the efficacy of Carboplatin in reducing the cellular viability of chemoresistant HGSC cells. (A–D) Bar charts showing cell viability of OVCAR8 and OVCAR8 PTX R C cells obtained by CellTiter-Glo® Luminescent assays after exposure to a fixed-dose ratio that corresponds to 0.25, 0.5, 1, 2, and 4 times the individual IC50 values of each drug. For 72 h, Carboplatin was combined with (A and B) Pitavastatin and (C and D) Ivermectin. The combined treatment was administered simultaneously. All assays were performed in triplicate in at least three independent experiments. The data are expressed as the mean ± standard deviation and plotted using GraphPad Prism Software Inc. v6 (GraphPad Software Inc., Boston, CA, USA). Statistical analysis was performed using ordinary one-way ANOVA followed by Šıdák’s multiple comparison test (A–D), with * *p* < 0.05, ** *p* < 0.001, *** *p* < 0.005, and **** *p* < 0.0001 considered statistically significant. HGSC, high-grade serous carcinoma; Figure S2: Combining Carboplatin with Pitavastatin has an antagonistic effect on OVCAR8 and OVCAR8 PTX R C cells for HSA, Bliss Independence, and ZIP synergy models. 2D and 3D synergy plots showed the drug synergism of OVCAR8 (A–C) and OVCAR8 PTX R C (D–F) cells after exposure to fixed-dose ratios that correspond to 0.25, 0.5, 1, 2, and 4 times the individual IC50 values of each drug. Carboplatin was combined with Pitavastatin for 72 h. The combined treatment was administered simultaneously. All assays were performed in triplicate in at least three independent experiments. The synergy scores were categorized as follows: <−10 (antagonism, green), −10 to 10 (additivity, white), and >10 (synergism, red). HSA, high single agent; ZIP, zero interaction potency; Figure S3: Combining Carboplatin with Ivermectin has an antagonistic effect on OVCAR8 and OVCAR8 PTX R C cells for HSA, Bliss Independence, and ZIP synergy models. 2D and 3D synergy plots showed the drug synergism of OVCAR8 (A–C) and OVCAR8 PTX R C (D–F) cells after exposure to fixed-dose ratios that correspond to 0.25, 0.5, 1, 2, and 4 times the individual IC50 values of each drug. Carboplatin was combined with Pitavastatin for 72 h. The combined treatment was administered simultaneously. All assays were performed in triplicate in at least three independent experiments. The synergy scores were categorized as follows: <−10 (antagonism, green), −10 to 10 (additivity, white), and >10 (synergism, red). HSA, high single agent; ZIP, zero interaction potency; Figure S4: Pitavastatin and Ivermectin increase the efficacy of Paclitaxel in reducing the cellular viability of chemoresistant HGSC cells. (A–D) Bar charts showing cell viability of OVCAR8 and OVCAR8 PTX R C cells obtained by CellTiter-Glo® Luminescent assays after exposure to a fixed-dose ratio that corresponds to 0.25, 0.5, 1, 2, and 4 times the individual IC50 values of each drug. For 72 h, Carboplatin was combined with (A and B) Pitavastatin and (C and D) Ivermectin. The combined treatment was administered simultaneously. All assays were performed in triplicate in at least three independent experiments. The data are expressed as the mean ± standard deviation and plotted using GraphPad Prism Software Inc. v6 (GraphPad Software Inc., Boston, CA, USA). Statistical analysis was performed using ordinary one-way ANOVA followed by Šıdák’s multiple comparison test (A–D), with * *p* < 0.05, ** *p* < 0.001, *** *p* < 0.005, and **** *p* < 0.0001 considered statistically significant. HGSC, high-grade serous carcinoma; Figure S5: Combining Paclitaxel with Pitavastatin has an antagonistic effect on OVCAR8 and OVCAR8 PTX R C cells for HSA, Bliss Independence, and ZIP synergy models. 2D and 3D synergy plots showed the drug synergism of OVCAR8 (A–C) and OVCAR8 PTX R C (D–F) cells after exposure to fixed-dose ratios that correspond to 0.25, 0.5, 1, 2, and 4 times the individual IC50 values of each drug. Carboplatin was combined with Pitavastatin for 72 h. The combined treatment was administered simultaneously. All assays were performed in triplicate in at least three independent experiments. The synergy scores were categorized as follows: <−10 (antagonism, green), −10 to 10 (additivity, white), and >10 (synergism, red). HSA, high single agent; ZIP, zero interaction potency; Figure S6: Combining Paclitaxel with Ivermectin has an antagonistic effect on OVCAR8 and OVCAR8 PTX R C cells for HSA, Bliss Independence, and ZIP synergy models. 2D and 3D synergy plots showed the drug synergism of OVCAR8 (A–C) and OVCAR8 PTX R C (D–F) cells after exposure to fixed-dose ratios that correspond to 0.25, 0.5, 1, 2, and 4 times the individual IC50 values of each drug. Carboplatin was combined with Pitavastatin for 72 h. The combined treatment was administered simultaneously. All assays were performed in triplicate in at least three independent experiments. The synergy scores were categorized as follows: <−10 (antagonism, green), −10 to 10 (additivity, white), and >10 (synergism, red). HSA, high single agent; ZIP, zero interaction potency.
