# Supplementary material for: Ivermectin Strengthens Paclitaxel Effectiveness in High-Grade Serous Carcinoma in 3D Cell Cultures
Source: Pharmaceuticals (Basel). 2024 Dec 25;18(1):14. doi: 10.3390/ph18010014 (PMC11769219; doi:10.3390/ph18010014)
Supplement: Supplementary file 1 [file pharmaceuticals-18-00014-s001.zip › pharmaceuticals-3361115-supplementary.pdf]

Supplementary Material

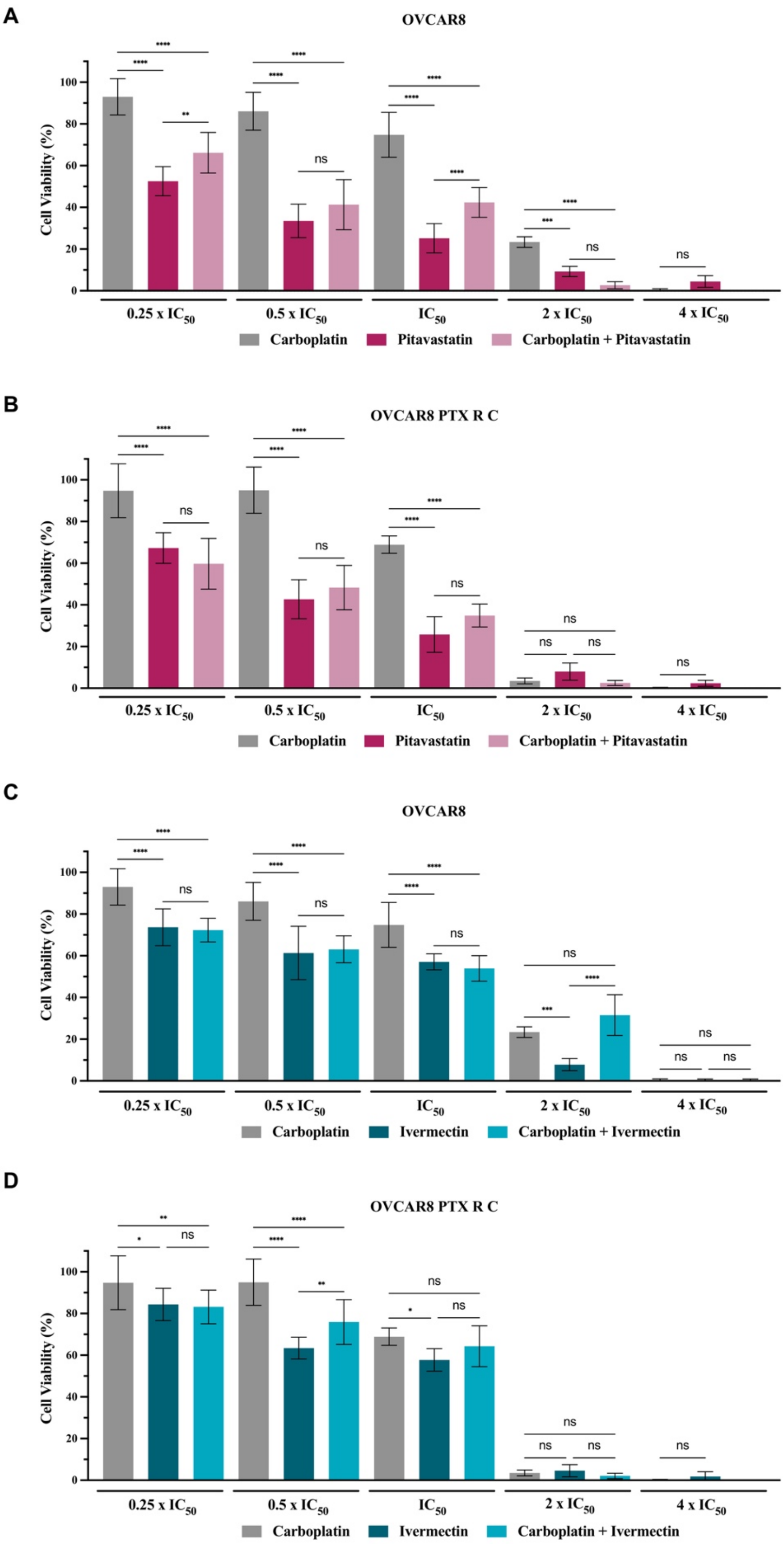

Figure S1. Pitavastatin and Ivermectin increase the efficacy of Carboplatin in reducing the cellular viability of chemoresistant HGSC cells. (**A**–**D**) Bar charts showing cell viability of OVCAR8 and OVCAR8 PTX R C cells obtained by CellTiter-Glo® Luminescent assays after exposure to a fixed-dose ratio that corresponds to 0.25, 0.5, 1, 2, and 4 times the individual IC_{50} values of each drug. For 72 hours, Carboplatin was combined with (**A** and **B**) Pitavastatin and (**C** and **D**) Ivermectin. The combined treatment was administered simultaneously. All assays were performed in triplicate in at least three independent experiments. The data are expressed as the mean ± standard deviation and plotted using GraphPad Prism Software Inc. v6 (GraphPad Software Inc. Boston, CA, USA). Statistical analysis was performed using ordinary one-way ANOVA followed by Šıďák's multiple comparison test (**A**–**D**), with * $p < 0.05$, ** $p < 0.001$, *** $p < 0.005$, and **** $p < 0.0001$ considered statistically significant. HGSC, high-grade serous carcinoma.

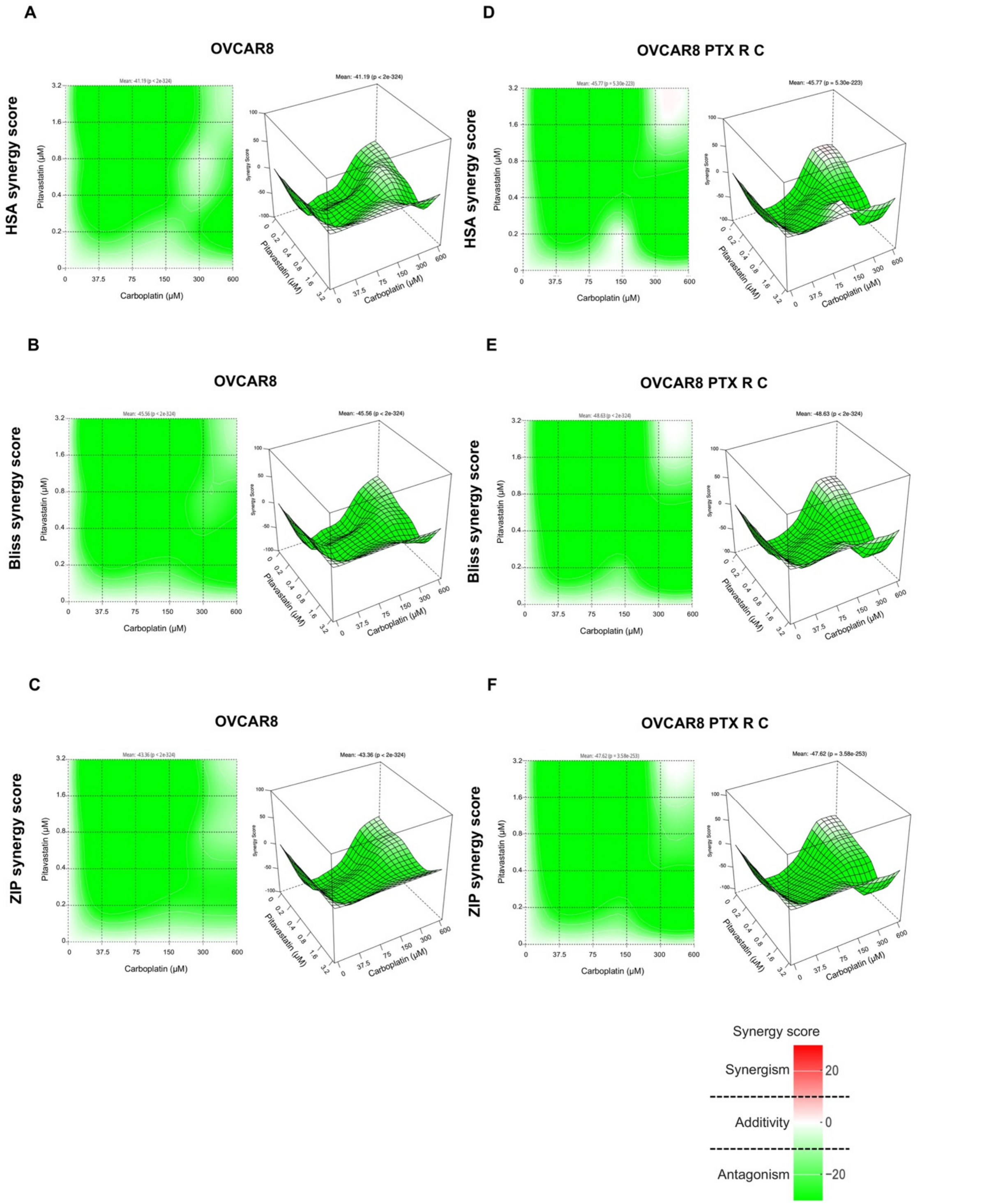

Figure S2. Combining Carboplatin with Pitavastatin has an antagonistic effect on OVCAR8 and OVCAR8 PTX R C cells for HSA, Bliss Independence, and ZIP synergy models. 2D and 3D synergy plots showed the drug synergism of OVCAR8 (**A**–**C**) and OVCAR8 PTX R C (**D**–**F**) cells after exposure to fixed-dose ratios that correspond to 0.25, 0.5, 1, 2, and 4 times the individual IC_{50} values of each drug. Carboplatin was combined with Pitavastatin for 72 hours. The combined treatment was administered simultaneously. All assays were performed in triplicate in at least three independent experiments. The synergy

scores were categorized as follows: <−10 (antagonism, green), −10 to 10 (additivity, white), and >10 (synergism, red). HSA, high single agent; ZIP, zero interaction potency.

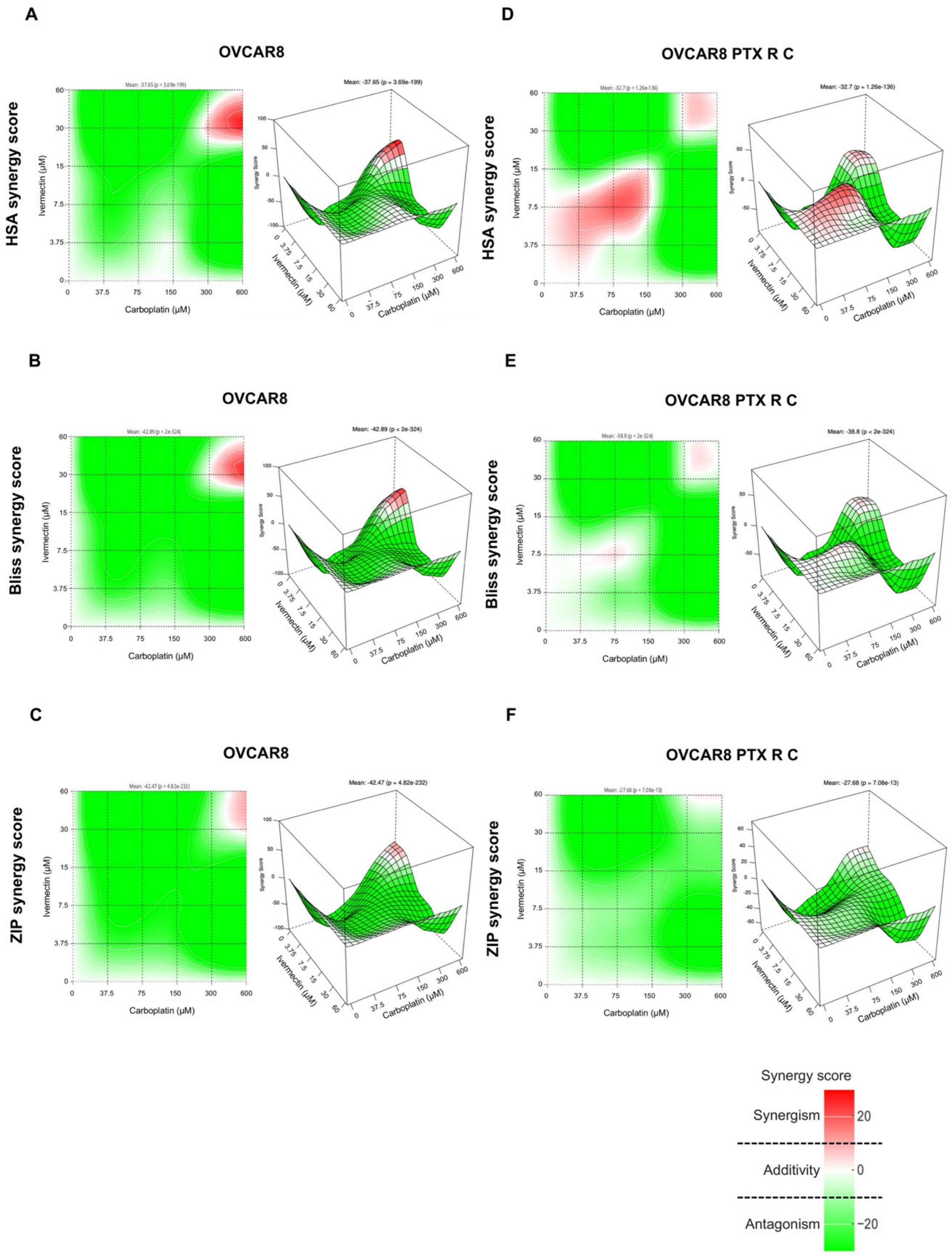

Figure S3. Combining Carboplatin with Ivermectin has an antagonistic effect on OVCAR8 and OVCAR8 PTX R C cells for HSA, Bliss Independence, and ZIP synergy models. 2D and 3D synergy plots showed the drug synergism of OVCAR8

(**A**–**C**) and OVCAR8 PTX R C (**D**–**F**) cells after exposure to fixed-dose ratios that correspond to 0.25, 0.5, 1, 2, and 4 times the individual IC_{50} values of each drug. Carboplatin was combined with Pitavastatin for 72 hours. The combined treatment was administered simultaneously. All assays were performed in triplicate in at least three independent experiments. The synergy scores were categorized as follows: <−10 (antagonism, green), −10 to 10 (additivity, white), and >10 (synergism, red). HSA, high single agent; ZIP, zero interaction potency.

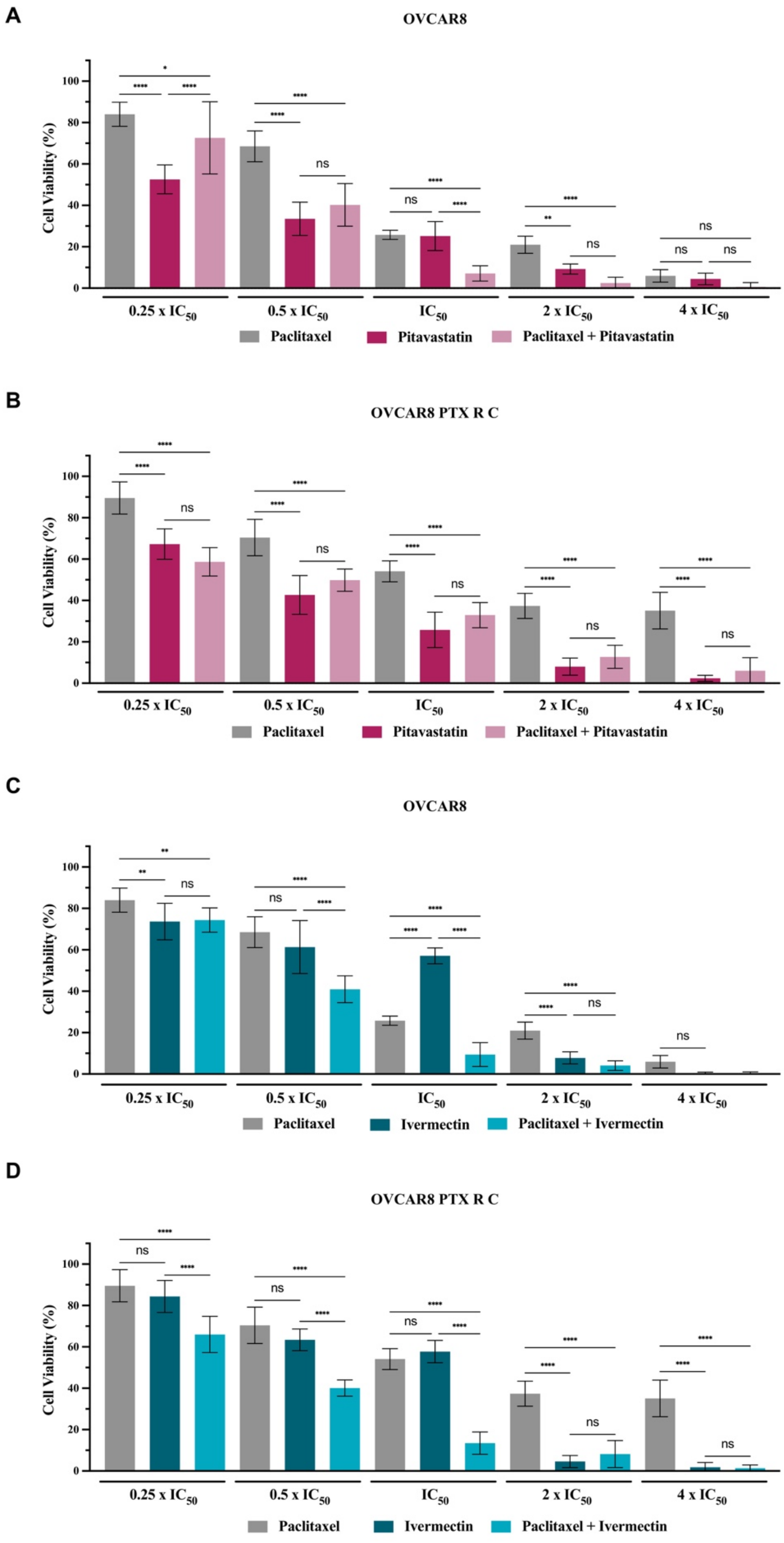

Figure S4. Pitavastatin and Ivermectin increase the efficacy of Paclitaxel in reducing the cellular viability of chemoresistant HGSC cells. (**A**–**D**) Bar charts showing cell viability of OVCAR8 and OVCAR8 PTX R C cells obtained by

CellTiter-Glo® Luminescent assays after exposure to a fixed-dose ratio that corresponds to 0.25, 0.5, 1, 2, and 4 times the individual IC_{50} values of each drug. For 72 hours, Carboplatin was combined with (**A** and **B**) Pitavastatin and (**C** and **D**) Ivermectin. The combined treatment was administered simultaneously. All assays were performed in triplicate in at least three independent experiments. The data are expressed as the mean ± standard deviation and plotted using GraphPad Prism Software Inc. v6 (GraphPad Software Inc. Boston, CA, USA). Statistical analysis was performed using ordinary one-way ANOVA followed by Šıďák's multiple comparison test (**A**–**D**), with * $p < 0.05$, ** $p < 0.001$, *** $p < 0.005$, and **** $p < 0.0001$ considered statistically significant. HGSC, high-grade serous carcinoma.

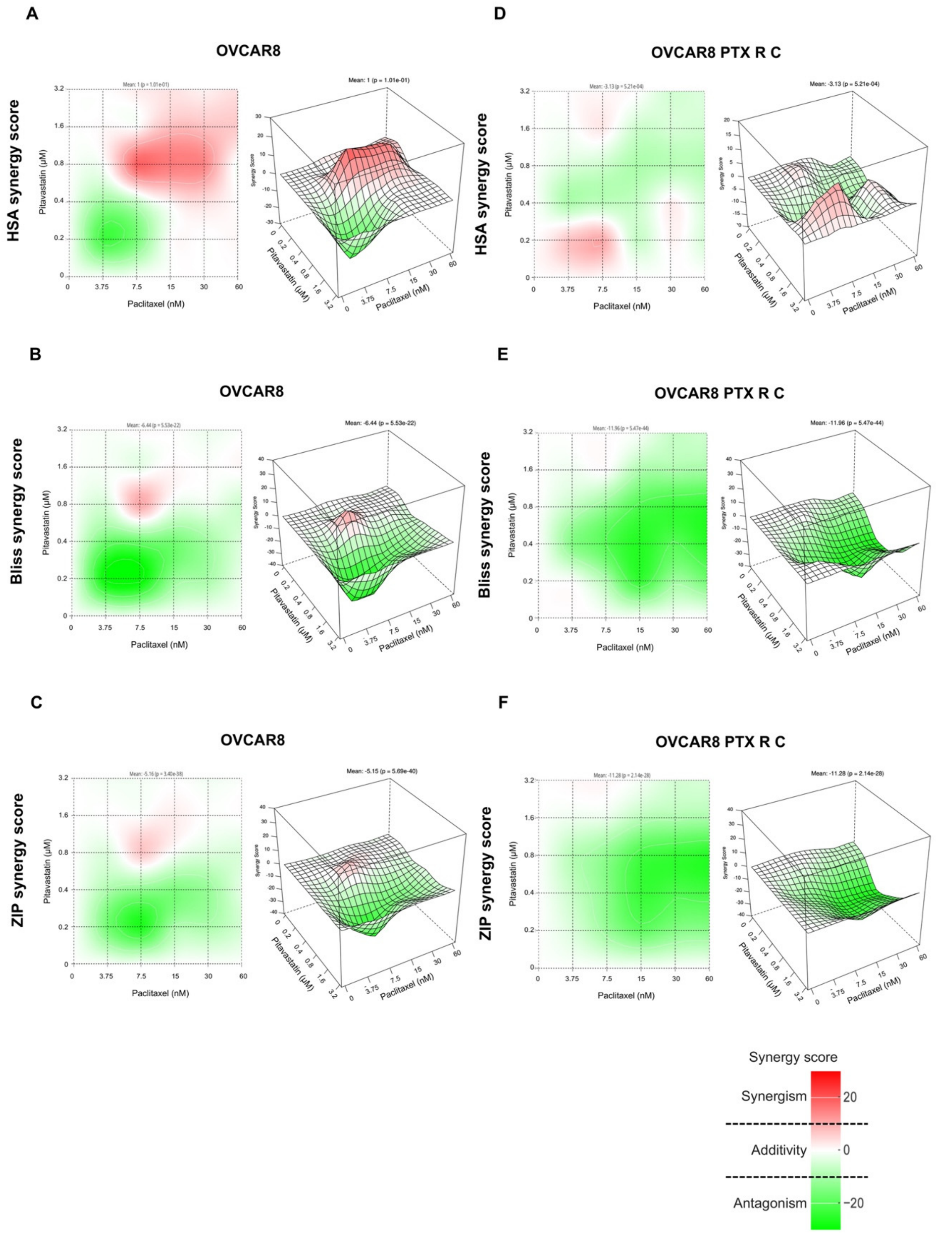

Figure S5. Combining Paclitaxel with Pitavastatin has an antagonistic effect on OVCAR8 and OVCAR8 PTX R C cells for HSA, Bliss Independence, and ZIP synergy models. 2D and 3D synergy plots showed the drug synergism of OVCAR8 (**A**–**C**) and OVCAR8 PTX R C (**D**–**F**) cells after exposure to fixed-dose ratios that correspond to 0.25, 0.5, 1, 2, and 4 times the individual IC_{50} values of each drug. Carboplatin was combined with Pitavastatin for 72 hours. The combined treatment was administered simultaneously. All assays were performed in triplicate in at least three independent experiments. The synergy

scores were categorized as follows: <−10 (antagonism, green), −10 to 10 (additivity, white), and >10 (synergism, red). HSA, high single agent; ZIP, zero interaction potency.

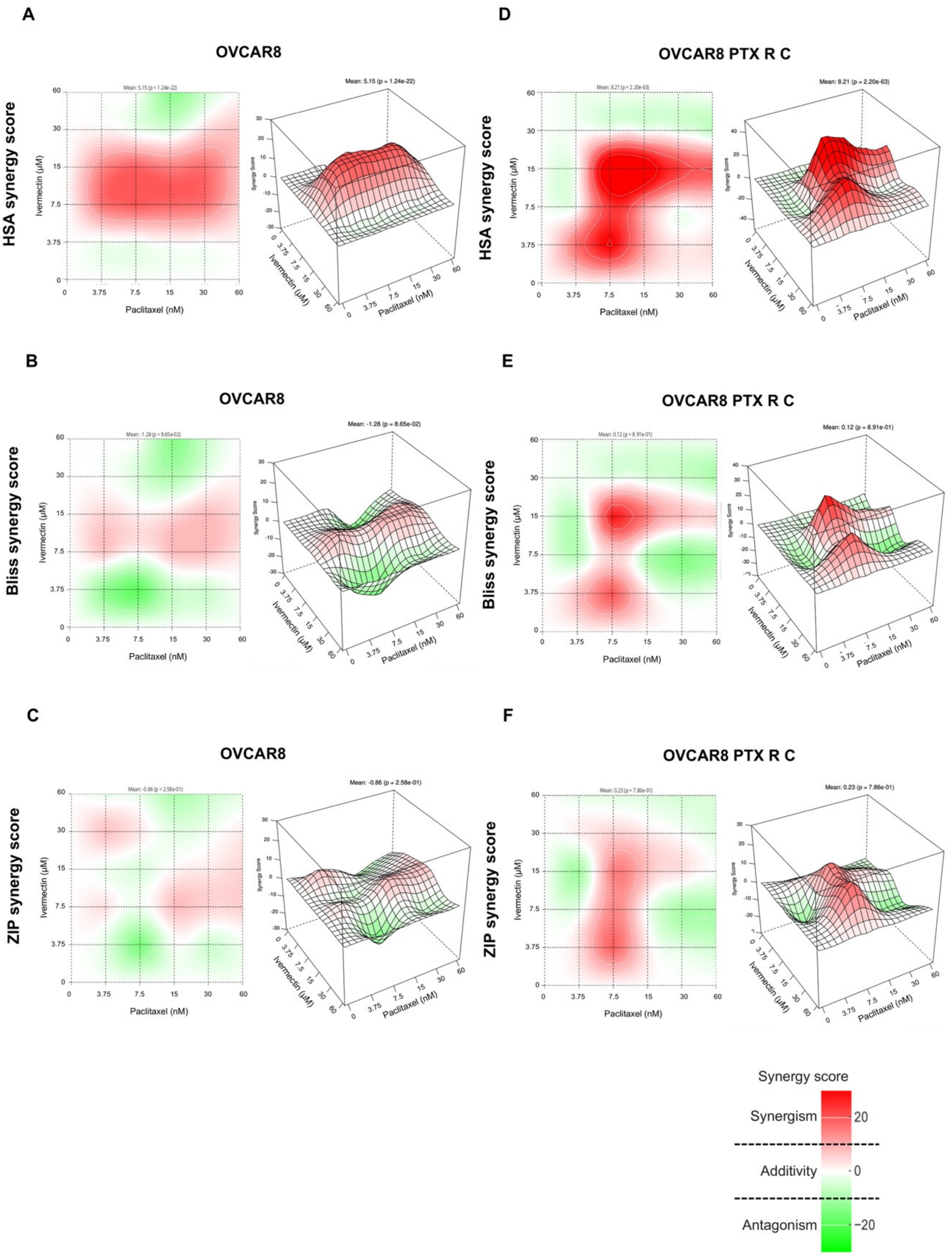

Figure S6. Combining Paclitaxel with Ivermectin has an antagonistic effect on OVCAR8 and OVCAR8 PTX R C cells for HSA, Bliss Independence, and ZIP synergy models. 2D and 3D synergy plots showed the drug synergism of OVCAR8 (**A**–

C) and OVCAR8 PTX R C (**D**–**F**) cells after exposure to fixed-dose ratios that correspond to 0.25, 0.5, 1, 2, and 4 times the individual IC_{50} values of each drug. Carboplatin was combined with Pitavastatin for 72 hours. The combined treatment was administered simultaneously. All assays were performed in triplicate in at least three independent experiments. The synergy scores were categorized as follows: <−10 (antagonism, green), −10 to 10 (additivity, white), and >10 (synergism, red). HSA, high single agent; ZIP, zero interaction potency.
